# Case Report: Ventriculoperitoneal Shunt Catheter Migration into the Gluteal Muscle

**DOI:** 10.3389/fped.2026.1848957

**Published:** 2026-06-03

**Authors:** Heng Zhao, Yufan Chen, Zhouwen Ni, Yang Zhao

**Affiliations:** Xinhua Hospital, School of Medicine, Shanghai Jiao Tong University, Shanghai, China

**Keywords:** fibrous encapsulation, gluteal muscle, Staphylococcus epidermidis biofilm, tethering effect, ventriculoperitoneal shunt migration

## Abstract

**Background and importance:**

Ventriculoperitoneal (VP) shunting remains the standard of care for hydrocephalus but is associated with a high rate of complications, including infection and catheter migration. While distal migration into visceral cavities (e.g., bowel, bladder) or via patent congenital defects (e.g., scrotum) is well-documented, migration into somatic musculature is exceptionally rare. We report a novel mechanism where chronic, inadequately treated colonization led to fibrotic fixation and subsequent traumatic migration into the gluteal muscle.

**Clinical presentation:**

A 3-year-old boy with Dandy-Walker malformation underwent VP shunting. One month postoperatively, he developed symptoms of shunt infection. Due to family refusal of shunt externalization, he was treated with prolonged courses of intravenous antibiotics (meropenem and linezolid), resulting in symptom resolution but likely persistent colonization. One year later, following a minor trauma, he presented with a painful mass in the left buttock. Imaging revealed the distal catheter had migrated through the pelvic outlet into the gluteus maximus. Laparoscopy confirmed the catheter was encased in a dense fibrous sheath. Cultures grew Staphylococcus epidermidis. The patient underwent hardware removal, external drainage, and subsequent shunt replacement with full recovery.

**Conclusion:**

This case illustrates that non-standard management of shunt infections—specifically relying on antibiotics without hardware removal—can lead to chronic biofilm formation and dense intraperitoneal fibrosis. This “tethering effect” transforms the flexible catheter into a rigid tract, predisposing it to mechanical migration into adjacent somatic structures upon trauma. Strict adherence to standardized protocols involving shunt externalization is critical to prevent such rare mechanical sequelae.

## Introduction

Ventriculoperitoneal (VP) shunt placement is the most common neurosurgical procedure for the management of pediatric hydrocephalus. Despite its efficacy, the procedure carries a significant complication profile, with failure rates reported as high as 30%–40% within the first year (1, 2). Common complications include mechanical obstruction, infection, and overdrainage (3).

Distal catheter migration is a well-recognized but less frequent complication, accounting for approximately 10% of shunt failures (4). The literature extensively documents migration into visceral cavities, such as the bowel (5–7), bladder (8, 9), and vagina (10), often driven by physiological peristalsis or erosion due to chronic pressure. Additionally, migration into the scrotum via a patent processus vaginalis is a known entity in infants (11, 12). Rare cases of supradiaphragmatic migration into the thorax (13–15), heart (16), or even the pulmonary artery (17) have also been described.

However, migration into somatic musculature is historically rare. While extrusion through the abdominal wall has been reported (18, 19), migration specifically into the gluteal region is exceptional and poorly understood (20).

Optimal management of shunt infection mandates the removal of the colonized hardware and temporary external ventricular drainage (EVD) (21, 22). Deviations from this standard can suppress acute symptoms while allowing biofilm formation—particularly by Staphylococcus epidermidis—to persist (23, 24). This report describes a previously undocumented mechanism in which chronic colonization led to intraperitoneal fibrotic fixation (tethering) of the distal catheter (25, 26). This fixation transformed a flexible catheter into a rigid vector that, following trauma, penetrated the pelvic floor and migrated into the gluteal muscle.

## Case presentation

### History and initial course

A 2-year-old boy diagnosed with Dandy-Walker malformation and progressive hydrocephalus underwent VP shunt placement following a failed cyst fenestration procedure. The initial postoperative course was uncomplicated.

### Infectious complications and management

Twenty-one days postoperatively, the patient developed fever and localized swelling over the abdominal incision. Cerebrospinal fluid (CSF) analysis via lumbar puncture demonstrated pleocytosis (WBC 22 × 10⁶/L), though initial cultures were negative. A diagnosis of presumptive shunt infection was made. Standard of care (shunt externalization) was recommended but refused by the family. The patient was treated empirically with meropenem, resulting in symptomatic improvement.

One month later, symptoms recurred with fever and abdominal pain. The family again declined surgical intervention. Based on antimicrobial sensitivities, a six-week course of meropenem and linezolid was administered. The patient's symptoms resolved, and inflammatory markers normalized. He was discharged and remained asymptomatic for approximately one year.

### Late presentation and migration

At 3 years of age, one year after the initial infection treatment, the patient presented with new-onset swelling and tenderness in the left buttock following a fall. Physical examination revealed a palpable, tender mass in the left gluteal region with overlying erythema (Figure 1). The patient was afebrile and neurologically intact.

**Figure 1:**
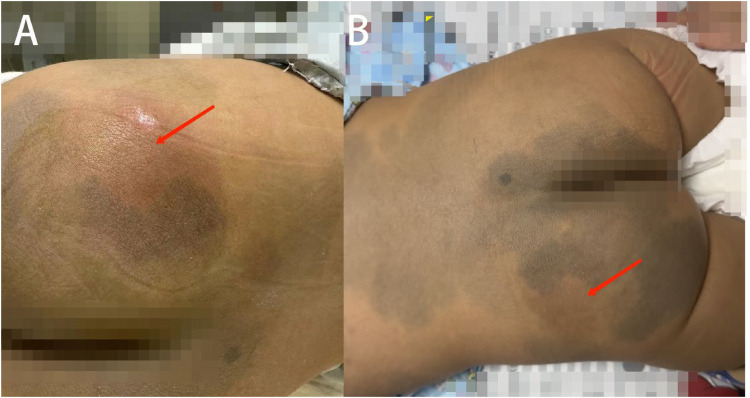
**(A)** Localized swelling and erythema over the left buttock (red arrow) at presentation, indicating the site of catheter migration. **(B)** Post-operative image showing resolution of the gluteal swelling.

### Radiological findings

Computed tomography (CT) and magnetic resonance imaging (MRI) of the pelvis demonstrated that the distal catheter tip had exited the peritoneal cavity, traversed the posterior pelvis, and penetrated the left gluteus maximus muscle (Figure 2). There was associated soft tissue edema but no distinct abscess collection initially visible.

**Figure 2:**
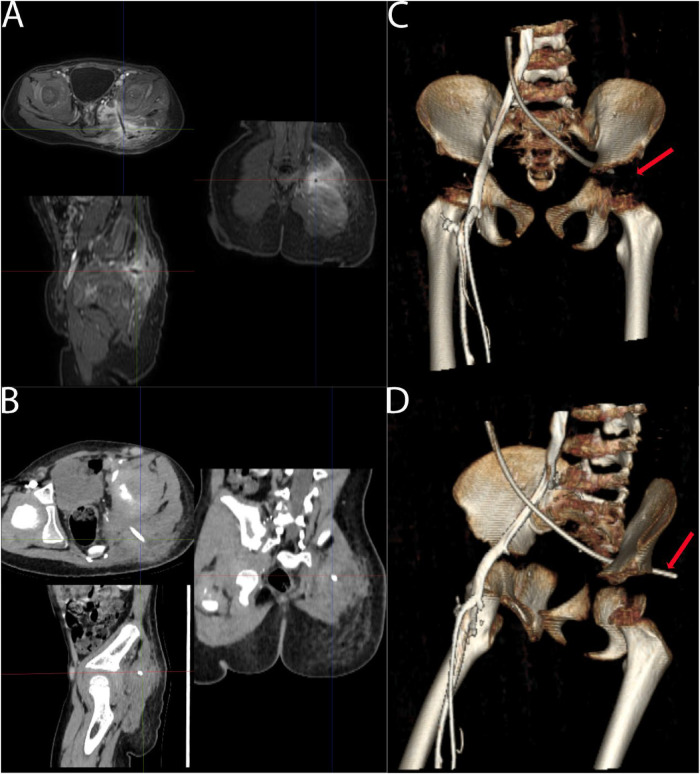
**(A)** Axial MRI sequence showing the linear hypointense tract of the catheter penetrating the gluteal muscle planes. **(B)** Sagittal CT reconstruction demonstrating the trajectory of the catheter extending from the pelvic cavity into the buttock soft tissue. **(C,D)** 3D reconstructed CT images visualizing the distal catheter tip position relative to the bony pelvis (red arrows), confirming extracompartmental migration.

### Surgical intervention and intraoperative findings

Due to the risk of skin breakdown, exploratory laparoscopy was performed. Intraoperative visualization revealed that the intraperitoneal portion of the catheter was not free-floating but was completely enveloped in a dense, vascularized fibrous sheath (Figure 3). This fibrosis formed a rigid tract extending through the posterior peritoneum into the pelvic outlet. The catheter was dissected from the adhesions and removed. CSF cultures obtained during surgery confirmed the growth of Staphylococcus epidermidis.

**Figure 3:**
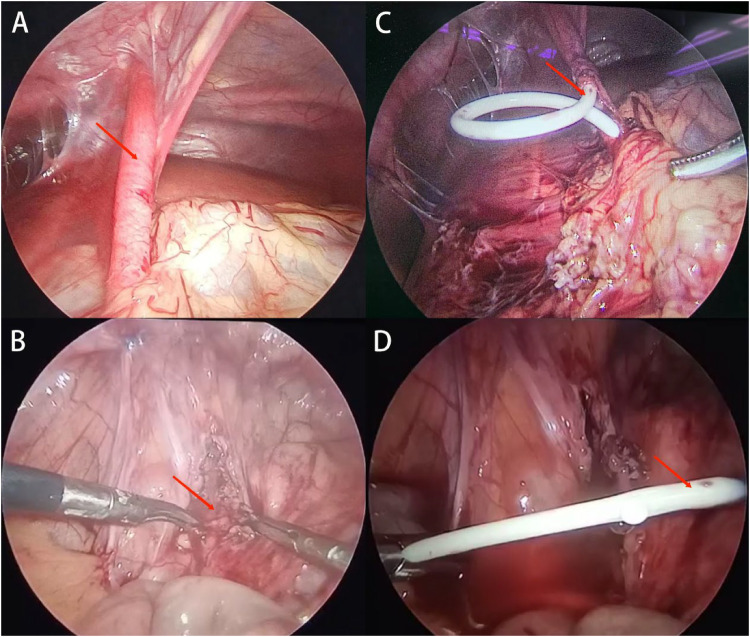
Intraoperative laparoscopic findings. **(A)** The distal peritoneal catheter is not free-floating but is encased in a dense, vascularized fibrous sheath (red arrow), confirming the “tethering” mechanism. **(B)** Surgical dissection of the fibrous adhesions to free the catheter. **(C,D)** The explanted catheter showing the integrity of the tubing, ruling out fracture as a cause of migration.

### Outcome

Following hardware removal and EVD placement, the patient completed a full course of intravenous antibiotics. Once CSF sterility was confirmed, a new VP shunt was placed. At 6-month follow-up, the patient remained complication-free (Table 1).

**Table 1:** Timeline of clinical progress and laboratory investigations.

Time point	Clinical status	CSF WBC count (10^6^/L)	CSF culture	Inflammatory markers (CRP/WBC)
T1: Postoperative Baseline (Days 1–7)	Uncomplicated recovery; afebrile	N/A	N/A	Normal
T2: Initial Infection Episode (Day 21)	Fever and localized abdominal swelling	22	Negative	Elevated
T3: Post-Antibiotic Course (Month 2)	Symptom resolution; clinical recovery	N/A	N/A	Normalized
T4: Late Migration Presentation (1 Year Later)	Gluteal mass and tenderness following minor trauma	Normal (Intraop)	Positive (S. epidermidis)	Normal

## Discussion

### Mechanisms of distal migration: a comprehensive review

Distal catheter migration is a distinct entity from disconnection or fracture. A review of the literature reveals diverse pathophysiological mechanisms depending on the anatomical site of migration (Table 2).

**Table 2:** Summary of rare distal catheter migrations of ventriculoperitoneal (VP) shunts.

Migration site	Proposed pathophysiology	Key references
Scrotum/Inguinal	“Windlass effect” via “Patent Processus Vaginalis (PPV).” Increased intra-abdominal pressure pushes the catheter into the patent hernial sac.	(11, 12)
Bowel/Anus	Chronic irritation and pressure necrosis lead to erosion into the bowel lumen (colon, stomach). Often asymptomatic (“silent”) until extrusion.	(5–7)
Bladder/Vagina	Erosion through the pouch of Douglas or bladder dome. Facilitated by stiffness of the catheter or local inflammation.	(8–10)
Thorax/Heart	Transdiaphragmatic migration via congenital hiatus (e.g., Bochdalek) or erosion. Negative intrathoracic pressure may “suck” the catheter upwards.	(13, 14, 16, 17)
Breast	Coiling of the tube creates a spring-like force; migration into breast implant capsules or tracking along subcutaneous planes.	(27, 28)
Abdominal Wall	Extrusion through previous incision sites or areas of weak fascia/necrosis, often secondary to superficial placement or rejection.	(18, 19)
Gluteal/Retroperitoneal	Fibrotic Fixation + Trauma (Current Case). Chronic infection causes dense fibrous encapsulation, creating a rigid tract that penetrates muscle upon trauma.	[Current Study]; (20)*

* Reference 21 describes a lumboperitoneal shunt migration, offering the closest anatomical parallel.

### The role of biofilm and fibrosis

The pathophysiology in our case is unique compared to the “erosion” (5, 6) or “peristaltic” (11) mechanisms described above. It is primarily driven by the host response to chronic Staphylococcus epidermidis colonization. S. epidermidis is the most common cause of shunt infection and is renowned for its ability to form biofilms on silicone catheters (23, 24).

In this case, the refusal of hardware removal allowed the biofilm to persist. While antibiotics suppressed the planktonic bacteria (resolving fever), the sessile bacteria within the biofilm continued to provoke a low-grade inflammatory response (29). This chronic inflammation stimulates the peritoneum to wall off the foreign body, leading to the formation of a dense, vascularized fibrous sheath (“abdominal pseudocyst” or “encapsulation”) (25, 26).

### The “tethering” effect

We propose the term “Tethering Effect” to describe the mechanical consequence of this fibrosis. Normally, a peritoneal catheter is free-floating and coils to dissipate kinetic energy during patient movement. However, the fibrous sheath observed in our case (Figure 3) effectively splinted the catheter, eliminating its flexibility. When the child fell, the force was transmitted directly through this rigid column. Unable to coil, the catheter acted as a spear, penetrating the path of least resistance—the pelvic outlet—and lodging in the gluteus muscle. This differs from the only other reported gluteal migration by Vassilev et al, (20) which involved a lumboperitoneal shunt tracking retroperitoneally.

### Pathophysiology of fibrotic tethering vs. abdominal pseudocysts

A distinctive feature of this case is the presence of a “fibrous sheath” rather than a classic abdominal pseudocyst (APC). While APCs are typically localized, fluid-filled collections of cerebrospinal fluid (CSF) contained by reactive peritoneum (23, 24), the laparoscopy in our patient revealed a dense, vascularized fibrous sleeve that encapsulated the catheter along its distal trajectory. This “tethering” pathology suggests a chronic inflammatory response likely driven by the subclinical Staphylococcus epidermidis colonization (25, 26). Unlike the expansive nature of a pseudocyst, this fibrosis essentially “rigidified” the flexible silicone catheter, transforming it into a semi-rigid rod (20).

### Implications for management

This case reinforces the Consensus Guidelines on the Management of Cerebrospinal Fluid Shunt Infection, which advocate for complete hardware removal (21, 22). Antibiotic suppression alone is associated with high failure rates and, as shown here, can lead to insidious mechanical complications due to fibrosis.

## Conclusion

Migration of a VP shunt catheter into the gluteal muscle is an exceedingly rare complication. We identified a “tethering” mechanism where chronic S. epidermidis colonization induced dense intraperitoneal fibrosis, fixing the catheter and predisposing it to traumatic penetration of somatic musculature. This highlights the critical importance of hardware removal in the management of shunt infections to prevent long-term fibrotic sequelae.

## Data Availability

The original contributions presented in the study are included in the article/Supplementary Material, further inquiries can be directed to the corresponding author.
